# Pitfall of Wide Wedge Resection: Risk of Overlooking Surgical Margin Shortage

**DOI:** 10.1093/icvts/ivag021

**Published:** 2026-01-13

**Authors:** Takuya Tokunaga, Shoichiro Morizono, Yuto Nonaka, Aya Takeda, Go Kamimura, Koki Maeda, Masaya Aoki, Toshiyuki Nagata, Koji Takumi, Hiroshi Kono, Hisashi Sahara, Kazuhiro Ueda

**Affiliations:** Department of General Thoracic Surgery, Surgery and Science, Graduate School of Medical and Dental Sciences, Kagoshima University, 8-35-1 Sakuragaoka, Kagoshima City, Kagoshima, 890-8520, Japan; Department of General Thoracic Surgery, Surgery and Science, Graduate School of Medical and Dental Sciences, Kagoshima University, 8-35-1 Sakuragaoka, Kagoshima City, Kagoshima, 890-8520, Japan; Department of General Thoracic Surgery, Surgery and Science, Graduate School of Medical and Dental Sciences, Kagoshima University, 8-35-1 Sakuragaoka, Kagoshima City, Kagoshima, 890-8520, Japan; Department of General Thoracic Surgery, Surgery and Science, Graduate School of Medical and Dental Sciences, Kagoshima University, 8-35-1 Sakuragaoka, Kagoshima City, Kagoshima, 890-8520, Japan; Department of General Thoracic Surgery, Surgery and Science, Graduate School of Medical and Dental Sciences, Kagoshima University, 8-35-1 Sakuragaoka, Kagoshima City, Kagoshima, 890-8520, Japan; Department of General Thoracic Surgery, Surgery and Science, Graduate School of Medical and Dental Sciences, Kagoshima University, 8-35-1 Sakuragaoka, Kagoshima City, Kagoshima, 890-8520, Japan; Department of General Thoracic Surgery, Surgery and Science, Graduate School of Medical and Dental Sciences, Kagoshima University, 8-35-1 Sakuragaoka, Kagoshima City, Kagoshima, 890-8520, Japan; Department of General Thoracic Surgery, Surgery and Science, Graduate School of Medical and Dental Sciences, Kagoshima University, 8-35-1 Sakuragaoka, Kagoshima City, Kagoshima, 890-8520, Japan; Department of Radiology, Graduate School of Medical and Dental Sciences, Kagoshima University, 8-35-1 Sakuragaoka, Kagoshima City, Kagoshima, 890-8520, Japan; Department of Biomaterials Science, Graduate School of Medical and Dental Sciences, Kagoshima University, 8-35-1 Sakuragaoka, Kagoshima City, Kagoshima, 890-8520, Japan; Division of Experimental Large Animal Research, Life Science and Laboratory Animal Research Unit, Center for Advanced Science Research and Promotion, Kagoshima University, 8-35-1 Sakuragaoka, Kagoshima City, Kagoshima, 890-8520, Japan; Department of General Thoracic Surgery, Surgery and Science, Graduate School of Medical and Dental Sciences, Kagoshima University, 8-35-1 Sakuragaoka, Kagoshima City, Kagoshima, 890-8520, Japan

**Keywords:** pulmonary parenchymal rupture, empty space, surgical margin, inflated CT, wedge resection, lung cancer

## Abstract

**Objectives:**

The recent increase in sublobar resections has been driven by favourable long-term outcomes and advances in stapling devices. However, maintaining an adequate resection margin remains a critical oncological requirement. This study aimed to investigate whether deep wedge resection induces subpleural alveolar injury that could lead to margin overestimation by creating undetectable internal cavities.

**Methods:**

We retrospectively analysed 33 consecutive patients who underwent wedge resection and CT imaging of resected lung specimens between December 2018 and February 2025. CT was performed on inflated specimens to better visualize internal lung architecture. We assessed the presence of an “empty space” adjacent to the staple line and correlated it with clinical factors, including depth of wedge resection (WR). Additionally, ex vivo porcine lung models were used to simulate deep WR, analyse compression effects, and identify histological damage caused by stapler compression.

**Results:**

CT imaging revealed empty spaces adjacent to the staple line in 10 of 33 specimens (30.3%), with a mean cavity length of 8.25 ± 3.2 mm. This artifact was significantly associated with deeper WR (≥26.3 mm) and increased stapler cartridge usage (median: 4 vs 3, *P* = .0298). In porcine experiments, compression to 2 mm thickness resulted in internal parenchymal rupture without pleural tearing, replicating the clinical findings.

**Conclusions:**

This study identified a potential mechanism by which deep wedge resection may lead to overestimation of the pathological margin due to stapler-induced parenchymal rupture. Further large-scale studies integrating oncological outcomes are warranted to clarify how wedge resection and segmentectomy should be appropriately selected for deep peripheral lung lesions.

## Graphical abstract

**Figure:**
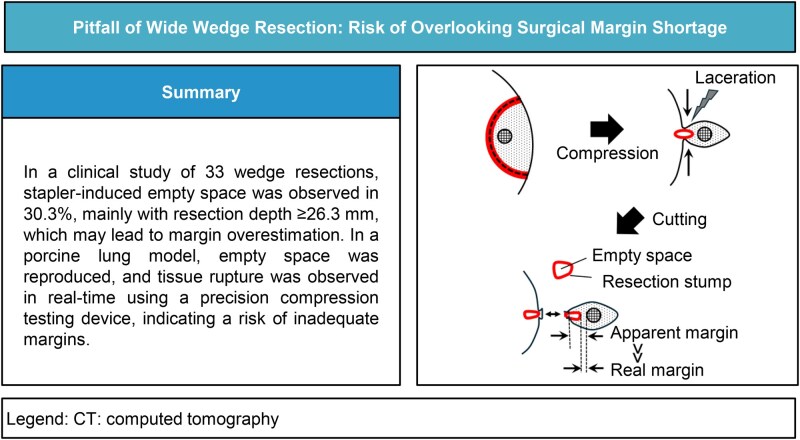

## INTRODUCTION

The long-term outcomes of sublobar resection for small peripheral non-small cell lung cancer (NSCLC) have been reported, leading to a growing acceptance of sublobar resection, including deep wedge resection.^1^^,^^2^ The widespread adoption of sublobar resection has been facilitated, in part, by advancements in surgical stapling devices, which now enable safe and reliable resection of even thick lung parenchyma without stapling failure. However, a fundamental prerequisite for sublobar resection to be oncologically acceptable is the achievement of an adequate surgical margin distance.^3–5^ Intraoperative palpation is commonly used to estimate the margin, but discrepancies are often observed between the margin distance assessed by palpation and the actual pathological margin. Several factors have been suggested to contribute to this discrepancy, including lung elasticity, the inability to detect microscopic invasion by palpation, and differences between the site of palpation and the pathological evaluation plane. To clarify these issues, we have been performing computed tomography (CT) scans of freshly resected, inflated lung specimens to evaluate the resection margin.^6^ Through the accumulation of cases, we identified a previously unrecognized mechanism that may cause significant discrepancies between palpation-based and pathological margin assessments. To investigate this hypothesis, we conducted compression testing and morphological evaluation using porcine lungs. In this study, we report a newly observed phenomenon that frequently occurs during deep wedge resection using surgical staplers. Recognizing this phenomenon is critical for preventing underestimation of margin distance and ensuring appropriate surgical margins. We believe this report is valuable in raising awareness among thoracic surgeons.

## METHODS

This retrospective observational study was conducted at Kagoshima University Hospital. The study protocol was approved by the Medical Research Ethics Committee of Kagoshima University (IRB approval no. 250133Epi; approved September 18, 2025). Written informed consent was waived because of the retrospective design; however, patients were given the opportunity to opt out via public notification.

### Clinical study

We retrospectively analysed 33 consecutive patients who underwent sublobar resection and postoperative CT imaging of resected lung specimens between December 2018 and February 2025. The sample size was determined by the number of eligible consecutive patients during the study period. No formal power calculation was performed because the aim was exploratory. The resected specimens were inflated by injecting air through a 23-gauge needle attached to a syringe, mimicking the in vivo lung inflation. Air was injected through the visceral pleura on the opposite side of the staple line to avoid direct disruption of the stapled area. The needle was advanced only shallowly, and inflation was performed gradually. Air easily leaked back through the puncture site, and the applied airway pressure was estimated to remain below approximately 10 cmH_2_O, which provided sufficient expansion for identifying internal cavities while minimizing the risk of overinflation. This method was applied consistently across all cases. The inflated specimens were then sealed in containers and scanned using computed tomography, as previously described.^6^

The following clinical and surgical parameters were collected: age, sex, pack-years smoked, severity of pulmonary emphysema on preoperative CT, side of surgery, tumour size, wedge resection type (resection including or not including the pulmonary sulcus), number of staplers used, and the distance from the lung surface to the resection margin (depth of wedge resection).

An endoscopic linear stapler was used, including the Endo GIA or Signia (Medtronic, Minneapolis, MN, United States), and the Powered Echelon Flex or Echelon 3000 (Ethicon, Cincinnati, OH, United States). The choice of cartridge/staple size was made at the surgeon’s discretion according to the intraoperative situation. The severity of emphysema was defined using quantitative CT analysis. DICOM data of the entire lung obtained from 1-mm-thick chest CT slices were analysed with an image-processing software, SYNAPSE VINCENT, version 6.8.0177 (Fujifilm, Tokyo, Japan). The total lung volume was extracted as the volume of voxels with attenuation values below −600 HU. Within this volume, the low-attenuation area (LAA) was defined as voxels below −950 HU. The percentage of LAA volume relative to the total lung volume was calculated and used as the severity of emphysema.

CT imaging of resected specimens was performed using the following parameters: tube voltage 80 kV, rotation speed 0.6 s/rotation, beam width 20 mm (0.625 mm × 32 mm), helical pitch 0.531, slice thickness 0.625 mm, slice interval 0.625 mm, scan field of view (FOV) 25.0 cm, matrix 512 × 512, reconstruction kernel HD Lung, and noise index (NI) 10.

### Ex vivo animal study

Ex vivo lungs of adult miniature pigs of approximately 50 kg were used. Although porcine lungs are smaller than human lungs, the relative resection depth and stapling configuration were selected to reproduce clinically relevant deep wedge resections in humans, particularly those requiring multiple cartridges and tighter compression at the deepest plane. Experiments (ii) and (iii) were performed using excised porcine lungs (*n* = 1 and 3, respectively) from miniature pigs that were euthanized immediately prior to tissue harvesting. For Experiments (i) and (iv), commercially available porcine lungs (*n* = 8 and 1, respectively) were used.

#### Experiment (i)

To simulate the deepest part of the resection plane during a deep wedge resection, lung parenchyma was slowly compressed using a bench vise to a thickness of 2.3 mm. After releasing the pressure, the lung was fixed by injecting 10% neutral buffered formalin through the airway. After 24 h, the specimen was sectioned, and the compressed area was macroscopically examined. Additionally, haematoxylin and eosin staining was performed to evaluate for structural disruption of the lung parenchyma. The target compression thickness of 2.3 mm was selected based on the minimal staple gap encountered during resection of the thickest lung parenchyma.

#### Experiment (ii)

To simulate a deep wedge resection in the left caudal lobe, a resection plane was delineated, and the deepest point of the lobe was compressed to 1.8 mm using a bench vise. This thickness corresponds to the compression achieved by the Purple cartridge of the Signia stapling system. An endotracheal tube was inserted into the left bronchus, and air was insufflated to inflate the left caudal lobe. CT imaging was then performed while maintaining the inflated state.

#### Experiment (iii)

An actual deep wedge resection of the right caudal lobe was performed using a 45 mm Signia Purple cartridge stapler. The resected specimen was then inflated and subjected to CT imaging. To exclude the possibility that empty space formation resulted from overinflation, in another porcine lung, the resected lung was examined before air insufflation. The staple line was sharply split perpendicular to the firing direction without applying external airway pressure.

#### Experiment (iv)

To simulate a deep wedge resection in the caudal lobe, a hypothetical resection line was established. Lung tissue along this line was compressed to a thickness of 2 mm using a precision compression testing device (TGE-5 kN, MinebeaMitsumi Inc., Nagano, Japan). The device continuously recorded the applied compression force and corresponding tissue thickness during stepwise compression. The raw data were automatically exported to Microsoft Excel. The relationship between tissue thickness and compression force was plotted using these exported data in Excel. As a reference, the porcine trachea was also compressed to 2 mm to compare its physical resistance.

### Statistical analysis

All statistical analyses were performed using EZR version 1.68 (Saitama Medical Center, Jichi Medical University). Continuous variables are presented as medians and ranges. Between-group comparisons of continuous variables were conducted using the Mann-Whitney *U* test, and categorical variables were compared using Fisher’s exact test. A 2-tailed *P*-value of <.05 was considered statistically significant. Due to the limited sample size, multivariable analyses were not performed in this study.

Receiver operating characteristic (ROC) curve analysis was performed with EZR to assess the ability of Depth of WR to discriminate between cases with and without empty space formation. The area under the curve (AUC) was calculated as a measure of diagnostic accuracy. ROC curve analysis was performed using the Youden index to determine the optimal cutoff value of resection depth for predicting empty-space formation.

## RESULTS

### Clinical study

**Figure 1.**
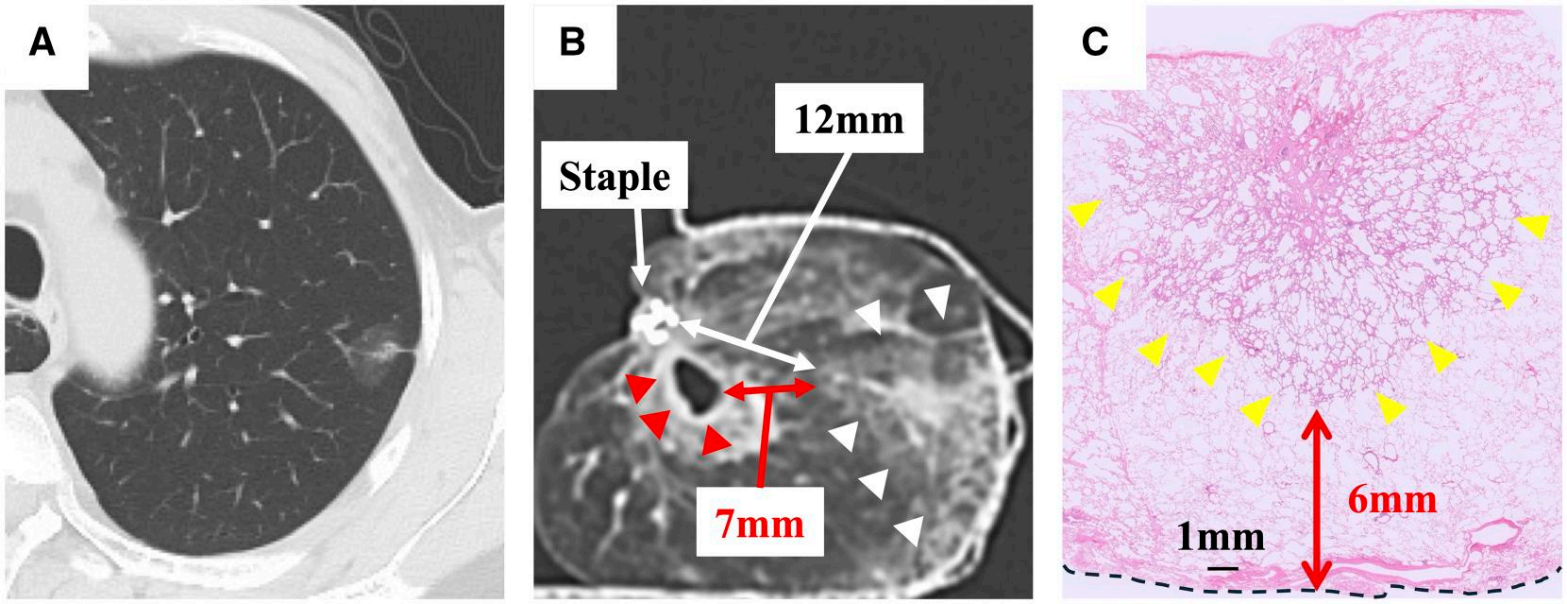
Representative Case. Chest computed tomography (CT) shows a 17-mm part-solid nodule in the apicoposterior segment of the left upper lobe (A). A CT image of the resected specimen, inflated with air after wedge resection, demonstrates the nodule and the stapled stump, which are 12 mm apart (white arrow) (B). An empty space (red arrowheads) is also seen adjacent to the stapled stump, located 7 mm from the nodule (white arrowheads). This corresponds well to the surgically confirmed margin on pathological examination (red arrow) (C). The upper side corresponds to the visceral pleura, and the dashed line represents the resection surface.

Among the 33 resected specimens evaluated by computed tomography, 10 specimens demonstrated a contiguous empty space adjacent to the staple line. Importantly, in all 10 cases, the internal cavity consistently appeared along the stapled parenchyma, independent of the puncture site. This observation indicates that the cavity was not created by air injection but was already present within the compressed tissue. A representative case is shown in **Figure 1**. Preoperatively, the lung parenchyma at the planned resection site appeared normal. However, in the CT image of the resected specimen, an empty space was observed in continuity with the resection margin. Although the tumour-to-margin distance on CT was 12 mm, the pathological margin was reported as 6 mm. This pathological distance closely matched the distance between the tumour and the edge of the empty space. The mean size of the observed empty space was 8.25 ± 3.2 mm.

The median age of the cohort was 68 years (range: 33-84 years), with 10 males and 23 females. Other background characteristics are summarized in **Table 1**.

**Table 1.** Patient Characteristics

Characteristics		*n *= 33
Age (years)		
Median (range)	68	(33-84)
Sex, *n* (%)		
Male	23	(69.7)
Female	10	(30.3)
PI (pack years)		
Median (range)	0	(0-64.5)
Severity of emphysema (%)		
Median (range)	3.1	(0-32.5)
Region, *n* (%)		
Right	21	(63.6)
Left	12	(36.3)
Type of WR, *n* (%)		
With sulcus	17	(51.5)
Without sulcus	16	(48.5)
Number of staplers used		
Median (range)	4	(2-7)
Empty space, *n* (%)		
Yes	10	(30.3)
No	23	(69.7)
Tumour size (mm)		
Median (range)	12.5	(4.7-24.5)
Depth of WR (mm)		
Median (range)	31.2	(20.2-67.8)

When comparing background factors between cases with and without empty space formation, the number of stapler cartridges used was significantly higher in the empty space group (median 4 vs 3; *P* = .0298). No significant differences were observed for age, sex, smoking history (pack-years), severity of emphysema on CT, side of resection, presence or absence of resection along the lung margin, tumour size, or depth of wedge resection (**Table 2**).

**Table 2.** Univariate Analysis of Factors Associated With Pulmonary Parenchymal Laceration

Factors	No empty space group		empty space group		*P*-value
	(*n *= 23)		*(n *= 10)		
Age (years)					
Median (range)	68	(33-84)	67	(53-79)	0.799
Sex, *n* (%)					
Male	15	(65.2)	8	(80)	0.682
Female	8	(34.8)	2	(20)	
PI (pack years)					
Median (range)	0	(0-64.5)	0	(0-30)	0.088
Severity of emphysema (%)					
Median (range)	4	(0-32.5)	3	(0-22.2)	0.571
Region, *n* (%)					
Right	14	(60.9)	7	(70)	0.710
Left	9	(39.1)	3	(30)	
Type of WR, *n* (%)					
With sulcus	11	(47.8)	5	(50)	1.000
Without sulcus	12	(52.2)	5	(50)	
Number of staplers used					
Median (range)	3	(2-5)	4	(3-7)	0.030
Tumour size (mm)					
Median (range)	12.5	(5.6-24.5)	13.0	(4.7-19.3)	0.811
Depth of WR (mm)					
Median (range)	26.8	(20.2-63.8)	39.9	(26.3-67.8)	0.053

To further explore the relationship between the Depth of WR and the presence of empty space, we performed a ROC analysis (**Figure S1**). The AUC was 0.727 (95% CI, 0.546-0.908; *P *= .012). Notably, no empty space was observed in cases with Depth of WR < 26.3 mm (*n* = 12), whereas in cases with Depth of WR ≥ 26.3 mm (*n* = 21), empty space formation was observed in 10 cases (47.6%).

### Ex vivo animal study

A notable finding from the compression tests was that although the pleura remained intact, rupture of the internal lung parenchyma was observed.

#### Experiment (i)

**Figure 2.**
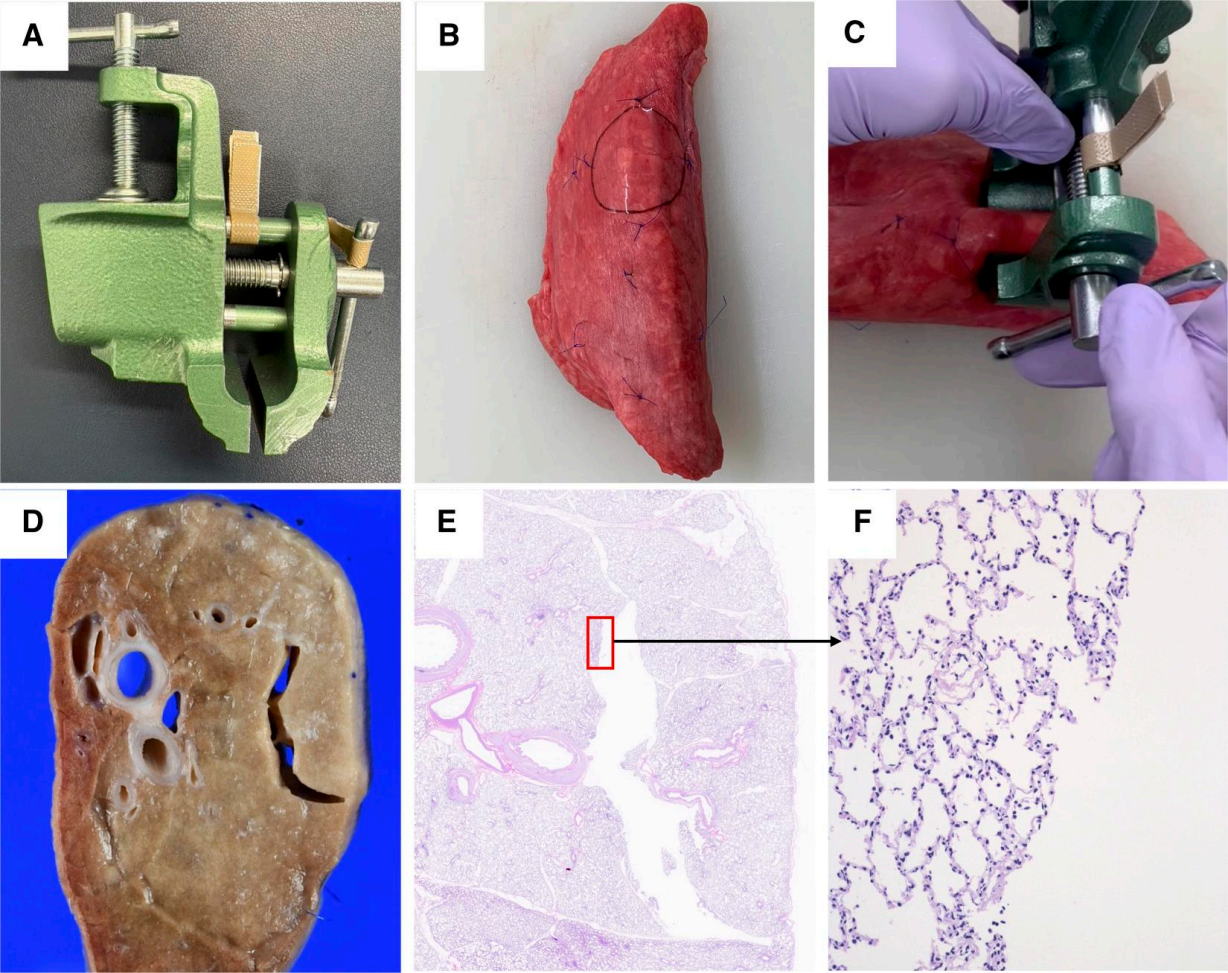
Experimental Findings in Porcine Lungs. A bench vise (A) was used to simulate lung compression with an endostapler. The porcine posterior lobe (B) was compressed to a thickness of 2.3 mm (C). An empty space (red arrow) was observed at the compression site both macroscopically (D) and microscopically (E). High-magnification views of the empty space (F) revealed rupture of the alveolar structures (black arrow) and tearing along the interlobular septa.

The lung parenchyma at the compression site showed visible rupture in the cut surface after formalin fixation, resulting in the formation of an empty space (**Figure 2D**). No pleural injury was identified. Haematoxylin and eosin staining revealed a mixture of alveolar rupture and tearing of the interlobular septa (**Figure 2E and F**).

#### Experiment (ii)

**Figure 3.**
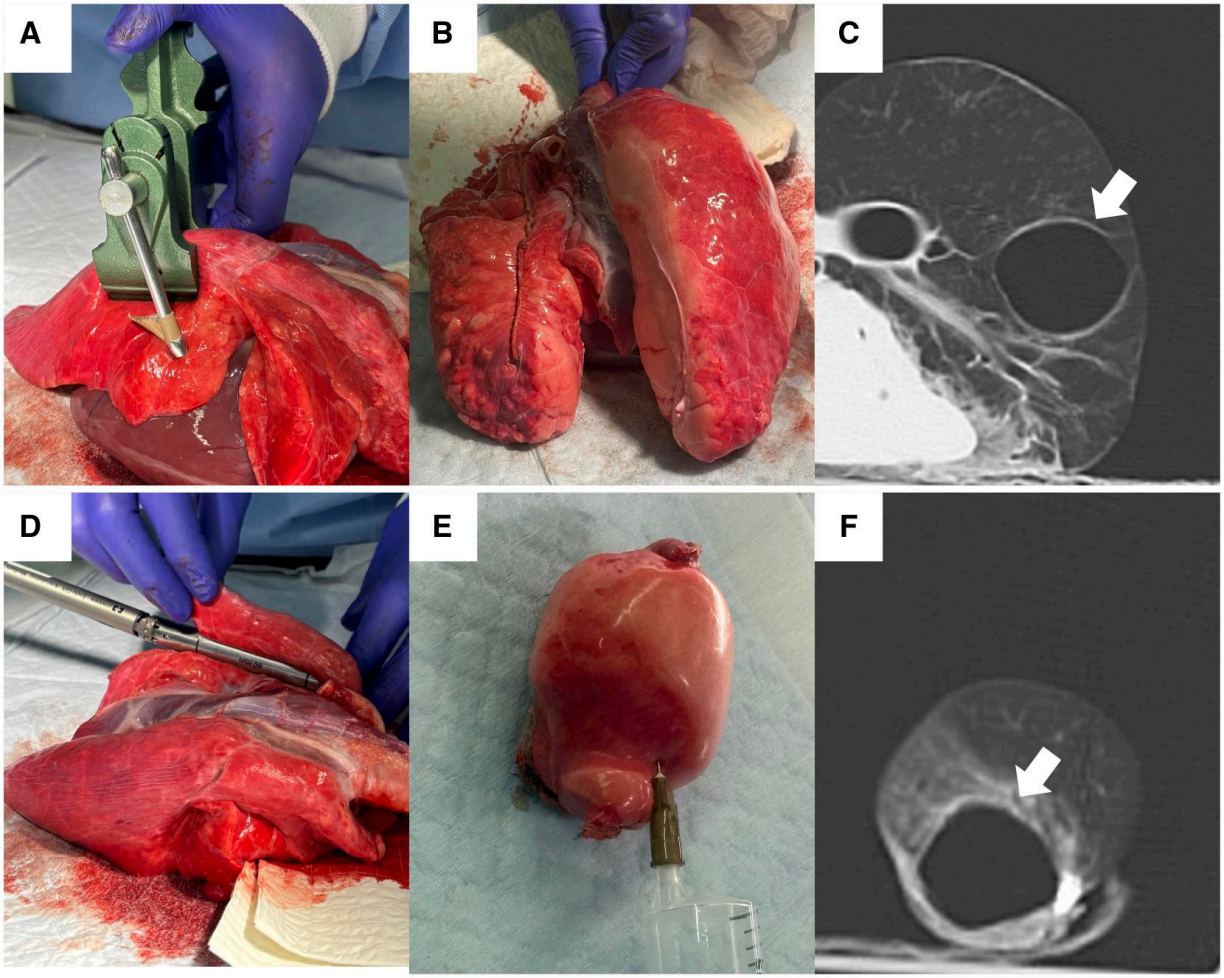
Experimental Findings in Porcine Lungs on Computed Tomography (CT). The posterior lobe of a porcine lung was compressed to a thickness of 1.8 mm (A) and then inflated with air using a ventilator (B). CT revealed an empty space (white arrow) at the compressed site (C). The posterior lobe of a porcine lung was stapled with cartridges of 1.8 mm thickness (D). After inflation of the resected specimen with air (E), CT scanning demonstrated an empty space (white arrow) adjacent to the stapled site (F).

CT imaging of the compressed lung showed that an empty space had formed exactly at the site of compression (**Figure 3C**).

#### Experiment (iii)

CT imaging of the inflated specimen following actual deep wedge resection using a Signia Purple 45 mm cartridge revealed an empty space contiguous with the resection margin (**Figure 3F**). Notably, a continuous empty space could be identified prior to any air inflation in porcine lung specimens, confirming that the empty space was not an overinflation artifact but was generated mechanically at the time of stapling (**Supplementary Video**).

#### Experiment (iv)

**Figure 4.**
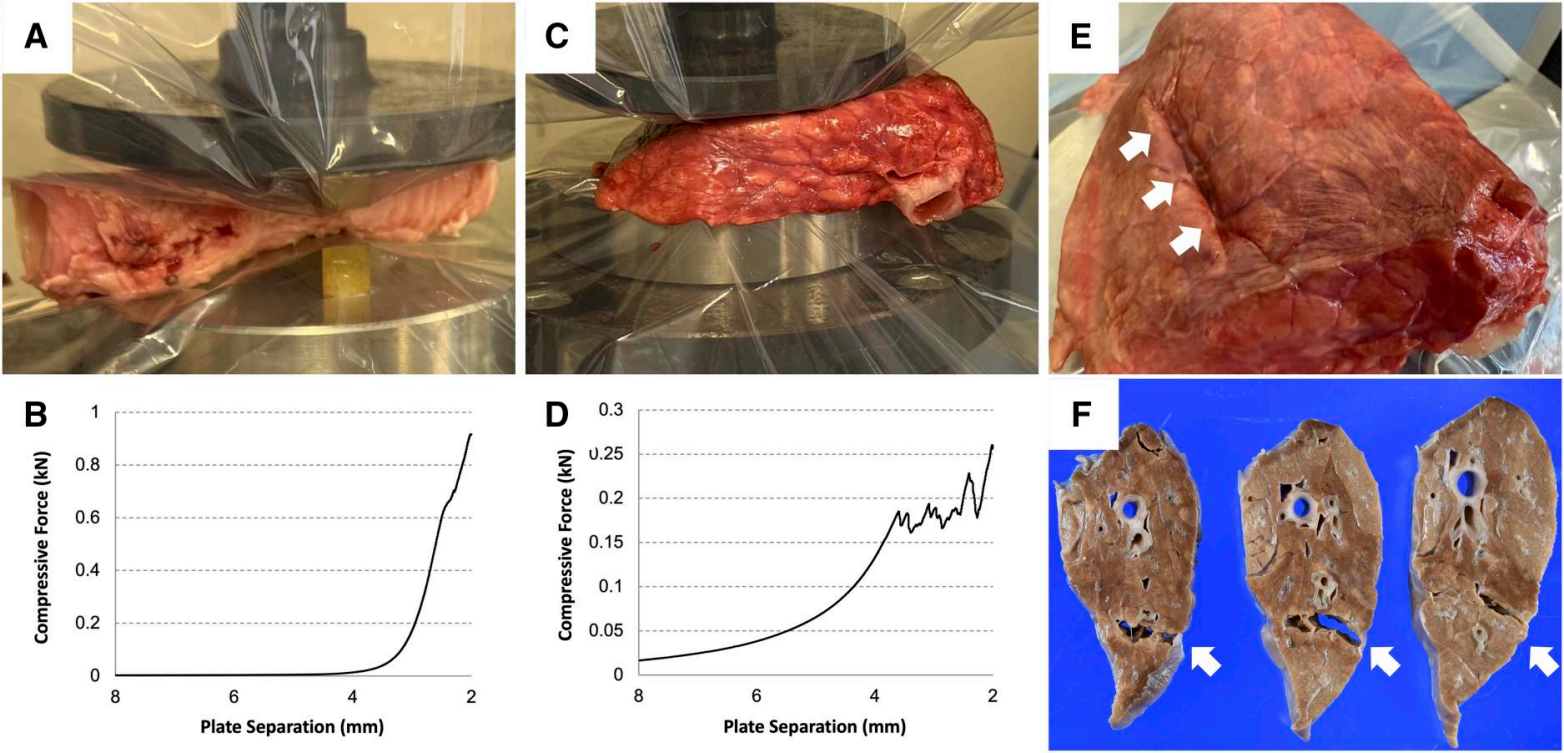
Experimental Compression Test Using a Precision Compression Testing Device Capable of Simultaneously Measuring Specimen Thickness and Applied Load in Real Time during Compression. When a porcine trachea was gradually compressed to a thickness of 2 mm (A), the applied load increased linearly with decreasing distance (B). In contrast, when a porcine lung was compressed to a thickness of 2 mm (C), the load increased in an accelerated manner, following a parabolic curve, and ceased to increase once the distance became less than 3.5 mm, suggesting rupture of the alveolar tissue (D). Compression to a thickness of 2 mm resulted in considerable deformation of the lung (white arrows) (E) and the formation of an empty space at the compression site (white arrows) (F).

When a porcine trachea was gradually compressed to a thickness of 2 mm (**Figure 4A**), the applied load increased linearly with decreasing thickness (**Figure 4B**). In contrast, compression of a porcine lung to the same thickness (**Figure 4C**) resulted in a non-linear, parabolic increase in load, followed by a plateau below 3.5 mm; rupture was observed when the tissue thickness reached approximately 3.5 mm. This value represents the onset of rupture under our test conditions and varies depending on the amount of tissue included between the compression plates. Compression to 2 mm thickness caused marked deformation of the lung tissue (**Figure 4E**), and an empty space was observed at the compression site (**Figure 4F**).

## DISCUSSION

In this study, we identified a previously underrecognized phenomenon: the formation of an “empty space” contiguous with the stapled stump following sublobar wedge resection. This space was observed in 10 of 33 (30.3%) clinical cases, and its presence was associated with a significantly greater number of stapler cartridges used. Notably, no empty spaces were seen in cases where the depth of wedge resection (WR) was less than 26.3 mm, whereas nearly half of the deeper resections exceeded this threshold and exhibited empty space formation. Our ex vivo experiments demonstrated that excessive mechanical compression can induce rupture of alveolar tissue without disruption of the visceral pleura, resulting in the formation of an artificial cavity at the site of maximal compression. Importantly, this cavity was already visible before any air was injected into the specimen, indicating that it was not caused by overinflation-induced separation or barotrauma-related artifact.

We hypothesize that in deep wedge resections, particularly those involving thick lung parenchyma, stapler compression may exceed the elastic limit of the alveolar framework. Once the compressive force surpasses the threshold (less than 3.5 mm thickness, as shown in our porcine model), mechanical failure of alveolar walls occurs, leading to tissue rupture internally but sparing the pleura. This results in the formation of an empty cavity that cannot be detected by intraoperative palpation, potentially causing overestimation of surgical margin length. This discrepancy was supported by our finding that radiologic margin measurements included the empty space, whereas the true histopathological margin was shorter.

The ex vivo compression test demonstrated that parenchymal rupture began when the tissue thickness decreased to approximately 3.5 mm. However, this threshold is not absolute and likely varies according to the volume of tissue engaged by the stapler jaws—smaller volumes may withstand compression to 2 mm, whereas larger volumes may rupture at greater thicknesses. Because commercial staplers compress tissue to a final thickness of 1.8-2.3 mm depending on cartridge type, these findings suggest that stapler-induced compression can readily exceed the mechanical tolerance of normal lung parenchyma, leading to internal tearing and formation of hidden cavities along the staple line.

While prior studies have documented discrepancies between palpated and histological margins in sublobar resection, the underlying mechanisms have largely been attributed to lung deflation, tissue handling, or microscopic spread.^7–9^ To our knowledge, no prior study has experimentally demonstrated parenchymal rupture induced by stapler compression as a source of artefactual empty space at the margin. Our findings add a novel mechanobiological explanation to this clinical problem.

This study highlights an important pitfall in margin assessment during sublobar resection, particularly when relying on palpation or intraoperative gross examination. Surgeons should be aware that deep wedge resections, especially those exceeding 26.3 mm in parenchymal depth, may result in the formation of an invisible cavity adjacent to the stapled margin. In such cases, margin distances estimated intraoperatively may be overestimated, and true oncologic safety may be compromised.

To mitigate this risk, careful case selection and surgical planning are essential. In cases where deep wedge resection would require multiple stapler cartridges—such as when the lesion is located deep within the parenchyma or is relatively large—margin overestimation due to empty space formation becomes more likely. For such lesions, segmentectomy may be a more appropriate surgical choice than wedge resection, ensuring both oncologic safety and accurate margin assessment. Therefore, rather than attempting to reduce the number of cartridges used in technically demanding cases, we propose that surgeons consider switching to anatomical segmentectomy in situations where deep parenchymal resections are anticipated.

Whether compression-induced rupture of the lung parenchyma occurs appears to depend on the relative relationship between the volume of tissue being compressed (ie, the extent of deep wedge resection) and the final compressed distance. Therefore, when compressing a large volume of lung tissue using a stapler, a cartridge with a wider closed staple height should be selected. However, the likelihood of parenchymal rupture is influenced by multiple factors. Host-related factors such as tissue fragility and alveolar wall thickness may also play a role and are likely to vary between individuals. It is possible that the radiographic translucency of lung parenchyma on CT may help predict the likelihood of empty space formation. However, in our analysis, neither the severity of emphysema nor the CT density of the compressed parenchyma (not shown in this report) correlated with the formation of empty space. In addition, the stapling technique itself may also influence the likelihood of rupture. It is generally recommended to staple slowly to ensure safe compression. Accordingly, all stapling in the present study was performed as slowly as feasible. To clarify these issues, accumulating data on compression test using clinical lung specimens may help define the individual limits of deep wedge resection that do not result in empty space formation.

**Figure 5.**
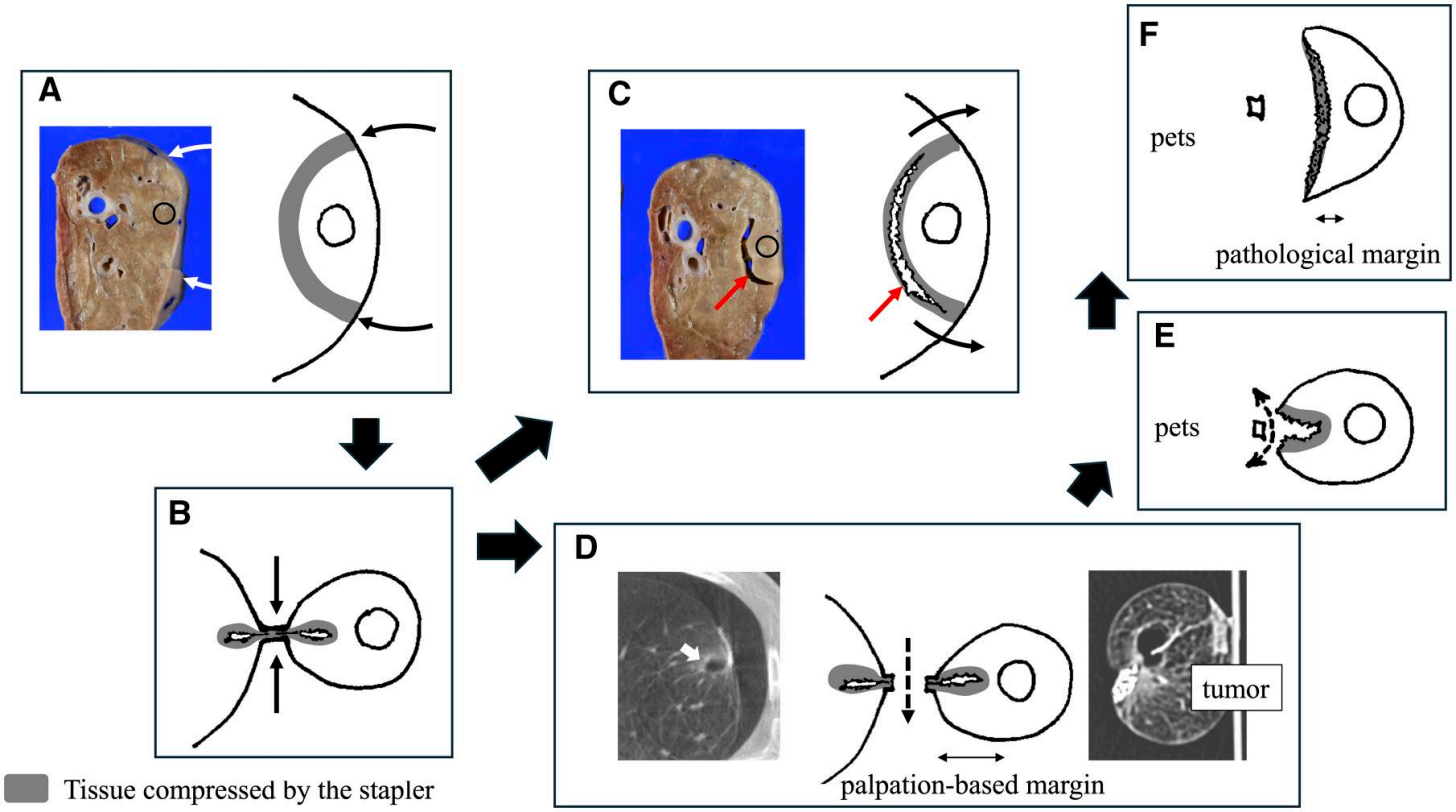
Mechanisms of Unexpected Margin Shortage after Sublobar Resection. Panel (A) illustrates the compression site (grey area) before lung compression. Further compression leads to laceration of the lung (B), resulting in the formation of an empty space at the compressed portion (C). After wedge resection (D), this empty space cannot be detected by palpation, although it can be visualized on CT, leading to overestimation of the margin distance. Once the staples are removed (E), the empty space becomes apparent, revealing an actual shortage of the margin distance (F).

The mechanism of lung rupture induced by compression is illustrated in **Figure 5A-D**. The underlying reason for the discrepancy between tactile assessment and pathological evaluation is also depicted (**Figure 5D-F**). Notably, the empty space created by compression and subsequent rupture of the lung parenchyma can be observed not only on the resected side of the staple line but also on the remaining lung side, as shown in **Figure 5D**. This finding was confirmed in our ex vivo experiments. In clinical practice, we occasionally perform intraoperative cone-beam CT imaging and have observed cases in which an empty space formed in the residual lung immediately after sublobar resection (**Figure S2**). In some patients, bullae develop near the staple line after lung resection, leading to persistent or delayed air leakage. Such stapling-related adverse events have been documented in clinical practice.^10^ The potential relationship between such adverse events and compression-induced space formation warrants further investigation. If a causal link is established, space formation may need to be recognized as a phenomenon to be avoided during deep wedge resection.

The frequency of sublobar resection has been increasing in recent years, partly due to the results of recent phase III clinical trials.^1^^,^^2^ However, segmentectomy has also been shown to be associated with a higher incidence of air-leak–related complications compared to lobectomy.^2^ Thus, there is a growing tendency to use staplers rather than electrocautery for intersegmental dissection during segmentectomy. With the advancement of stapling device technology, it has become feasible to compress and divide larger volumes of lung parenchyma simultaneously. Nevertheless, the potential for compression-induced empty space formation along staple lines can also occur during anatomical segmentectomy. This possibility warrants further investigation.

This study has several limitations. First, the relatively small sample size and low event rate (lack of power analysis) precluded multivariable analysis; therefore, the present findings should be interpreted as preliminary and hypothesis-generating rather than confirmatory. In particular, the ROC-derived cutoff of 26.3 mm for resection depth was obtained from a small cohort and should be regarded as supportive rather than definitive. External validation in larger, prospective datasets is required to determine whether this threshold is clinically meaningful. In addition, although deeper resections and empty space formation might theoretically increase the risk of air-leak–related complications, this relationship remains speculative and was not directly assessed in this study. However, to minimize the risk of stapler-induced parenchymal rupture, careful selection of stapler cartridge type and adjustment of compression angle or sequence of firing may help reduce excessive parenchymal strain. Future studies should explore these technical modifications and their impact on margin accuracy and postoperative air leaks. Second, because the optimal inflation pressure for detecting internal empty spaces has not been fully established, some small cavities may have remained undetected. Future studies with controlled and quantitative inflation protocols are required to standardize this procedure. Third, because porcine lungs have a smaller volume and different elasticity compared with human lungs, the findings should be interpreted as mechanistic rather than quantitatively generalizable. The model supports the mechanical origin of internal cavity formation but does not establish clinical thresholds for humans. Fourth, this study focused on identifying a mechanism underlying margin overestimation rather than evaluating oncological outcomes. Future studies linking this phenomenon to recurrence or survival are required to determine its clinical significance. Fifth, different stapler brands and cartridge types were used according to intraoperative availability, which may have contributed to variability in compression characteristics. Finally, this was a single-center study, which may limit the generalizability of the findings.

## CONCLUSIONS

The results of the ex vivo animal experiments suggest that stapler compression can mechanically induce internal parenchymal rupture, leading to overestimation of the surgical margin. While the findings support awareness of this phenomenon, they do not justify changing current surgical practice. Future multicentre studies incorporating oncological outcomes are needed to determine optimal indications and the balance between wedge resection and segmentectomy.

## Supplementary Material

ivag021_Supplementary_Data

## Data Availability

The data that support the findings of this study are available from the corresponding author on reasonable request.

## References

[1] Suzuki K , Watanabe S, Wakabayashi M, et al; West Japan Oncology Group and Japan Clinical Oncology Group. A single-arm study of sublobar resection for ground-glass opacity dominant peripheral lung cancer. J Thorac Cardiovasc Surg. 2022;163:289-301.e2. 33487427 10.1016/j.jtcvs.2020.09.146

[2] Saji H , Okada M, Tsuboi M, et al; West Japan Oncology Group and Japan Clinical Oncology Group. Segmentectomy versus lobectomy in small-sized peripheral non-small-cell lung cancer (JCOG0802/WJOG4607L): a multicentre, open-label, phase 3, randomised, controlled, non-inferiority trial. Lancet. 2022;399:1607-1617. 35461558 10.1016/S0140-6736(21)02333-3

[3] Nakagawa K , Watanabe S, Wakabayashi M, et al Risk factors for locoregional relapse after segmentectomy: supplementary analysis of the JCOG0802/WJOG4607L trial. J Thorac Oncol. 2025;20:157-166. 39395662 10.1016/j.jtho.2024.10.002

[4] Shiono S , Okumura T, Boku N, et al Outcomes of segmentectomy and wedge resection for pulmonary metastases from colorectal cancer. Eur J Cardiothorac Surg. 2017;51:ezw322-510. 10.1093/ejcts/ezw322 27773868

[5] Kim IH , Lee HP, Lee GD, et al Wedge resection versus segmentectomy in early-stage lung cancer considering resection margin and lymph node evaluation: a retrospective study. Eur J Cardiothorac Surg, 2025;67:ezaf281. 40875509 10.1093/ejcts/ezaf281

[6] Kamimura G , Ueda K, Suzuki S, et al Intraoperative computed tomography of a resected lung inflated with air to verify safety surgical margin. Quant Imaging Med Surg. 2022;12:1281-1289. 35111623 10.21037/qims-21-562 PMC8739102

[7] Kitazawa S , Bernards N, Gregor A, et al Feasibility of computed tomography-derived surgical margin assessment in an *ex vivo* sublobar lung resection model. Interdiscip Cardiovasc Thorac Surg. 2024;40:ivae211 39673785 10.1093/icvts/ivae211 PMC11706792

[8] Goldstein NS , Ferkowicz M, Kestin L, et al Wedge resection margin distances and residual adenocarcinoma in lobectomy specimens. Am J Clin Pathol. 2003;120:720-724. 14608898 10.1309/P47F-YW5U-4CRQ-0WFE

[9] Wolf A , Laskey D, Yip R, et al; IELCART Investigators. Measuring the margin distance in pulmonary wedge resection. J Surg Oncol. 2022;126:1350-1358. 35975701 10.1002/jso.27053

[10] Yano M , Iwata H, Hashizume M, et al Adverse events of lung tissue stapling in thoracic surgery. Ann Thorac Cardiovasc Surg. 2014;20:370-377. 24200667 10.5761/atcs.oa.13-00161

