# Supplementary material for: Pitfall of Wide Wedge Resection: Risk of Overlooking Surgical Margin Shortage
Source: Interdiscip Cardiovasc Thorac Surg. 2026 Jan 13;41(1):ivag021. doi: 10.1093/icvts/ivag021 (PMC12836424; doi:10.1093/icvts/ivag021)
Supplement: ivag021_Supplementary_Data [file ivag021_supplementary_data.zip › Supplementary Figure S1.pdf]

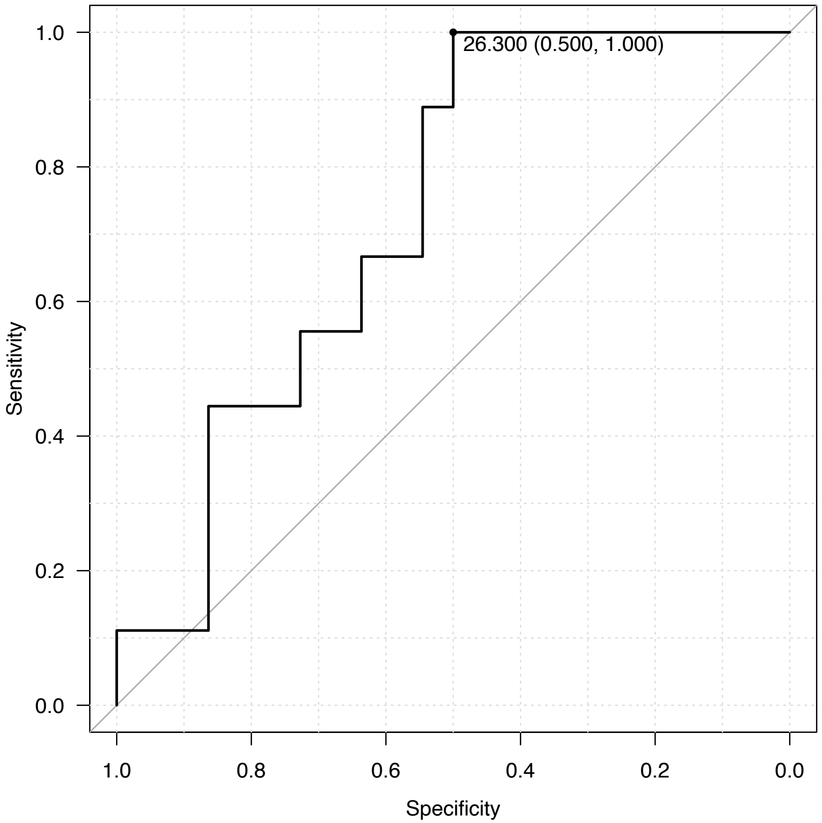

Supplementary Figure S1.

Receiver operating characteristic (ROC) curve for Depth of WR in relation to the presence of empty space. The area under the curve (AUC) was 0.727 (95% CI, 0.546–0.908; p = 0.012). The diagonal dotted line indicates an AUC of 0.5. The optimal threshold was determined by Youden's index.
